# Retinal Damage Induced by Internal Limiting Membrane Removal

**DOI:** 10.1155/2015/939748

**Published:** 2015-09-03

**Authors:** Rachel Gelman, William Stevenson, Claudia Prospero Ponce, Daniel Agarwal, John Byron Christoforidis

**Affiliations:** Department of Ophthalmology, University of Arizona Medical Center, Tucson, AZ 85711, USA

## Abstract

The internal limiting membrane (ILM), the basement membrane of the Müller cells, serves as the interface between the vitreous body and the retinal nerve fiber layer. It has a fundamental role in the development, structure, and function of the retina, although it also is a pathologic component in the various vitreoretinal disorders, most notably in macular holes. It was not until understanding of the evolution of idiopathic macular holes and the advent of idiopathic macular hole surgery that the idea of adjuvant ILM peeling in the treatment of tractional maculopathies was explored. Today intentional ILM peeling is a commonly applied surgical technique among vitreoretinal surgeons as it has been found to increase the rate of successful macular hole closure and improve surgical outcomes in other vitreoretinal diseases. Though ILM peeling has refined surgery for tractional maculopathies, like all surgical procedures it is not immune to perioperative risk. The essential role of the ILM to the integrity of the retina and risk of trauma to retinal tissue spurs suspicion with regard to its routine removal. Several authors have investigated the retinal damage induced by ILM peeling and these complications have been manifested across many different diagnostic studies.

## 1. Introduction

The internal limiting membrane (ILM) is the basal lamina of the inner retina that is formed by the footplates of Müller cells. It is the structural interface between the retina and the vitreous and is composed of collagen fibers, glycosaminoglycans, laminin, and fibronectin. The ILM is 1.5 *μ*m thick in the peripheral foveal area and is thickest in this region [1]. The ILM serves as a scaffold for cellular proliferation of myofibroblasts, fibrocytes, and retinal pigment epithelium (RPE) cells [2]. Experimental studies on embryonic mouse and chick eyes have shown that the ILM is a critical component of retinal histogenesis and optic axonal growth and navigation to the optic disc. Halfter et al. demonstrated that the absence of the ILM caused permanent retraction of the endfeet of neuroepithelial cells from the vitreal surface of the retina and the formation of a disorganized and abnormally thickened ganglion cell layer [3]. Despite its essential role in early retinal and optic nerve development, in pathologic conditions cellular proliferation on the ILM is strongly correlated with tractional forces on the retina; this association coupled with the tendency of the ILM to thicken with age makes ILM removal mandatory to relieve the contractile forces in tractional maculopathies. Furthermore, since ILM removal has also been found to decrease the risk of epiretinal membrane development postoperatively, the indications for its application are broadened to include several vitreoretinal conditions [4].

ILM peeling is now a widely recognized technique used routinely for traction maculopathies, but what are the possible complications of this intervention? It is a technique that requires additional intraoperative agents, instruments, and surgical time. No studies or reports to date have shown adverse visual outcomes in patients status after an ILM peel, but there has yet to be a large enough randomized control trial assessing side effects of ILM removal, and therefore the question remains: Does the ILM have a function vital to the integrity of the retina that would render it damage upon ILM removal? If so, what type of retinal damage can this surgical technique induce?

## 2. The History of ILM Peeling

ILM peeling is a surgical technique commonly used today to treat various vitreoretinal disorders including macular holes, macular puckers, epiretinal membranes, diabetic macular edema, retinal detachment, retinal vein occlusions, vitreomacular traction, optic pit maculopathy, and Terson syndrome [4]. It was not until the late 1980s when the possibility of ILM peeling was even considered to be a surgical option in the treatment of vitreoretinal disorders; in a 1989 pilot study, Kelly and Wendel performed vitrectomy and removal of the posterior cortical vitreous to relieve traction over the macula, shedding light on ILM peeling as a possible therapy in the treatment of full thickness macular holes. Prior to this, idiopathic macular holes were considered an untreatable condition [9]. Shortly following the pilot study, in the 1990s Morris et al. reported promising results of intentional ILM peeling in the treatment of hemorrhagic macular cysts due to Terson syndrome. Specifically, 83% of the study subjects' eyes had a visual acuity of 20/25 or better without development of observable reproliferation. With these favorable results, Morris et al. postulated ILM removal as a surgical technique that could be used for other tractional types of maculopathies [10].

## 3. Technique

**Figure 1:**
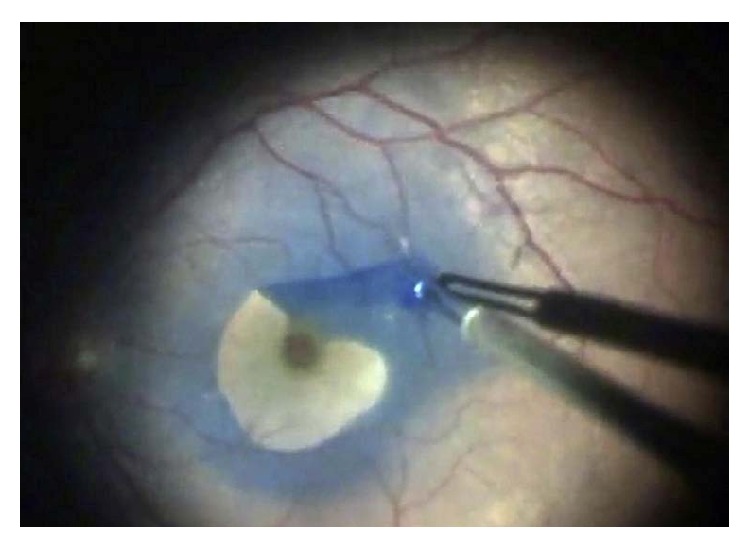
ILM peeling after staining with brilliant blue dye [5].

ILM peeling begins with pars plana vitrectomy and posterior hyaloid removal. Following these steps, adjuvant dyes are used to stain the translucent ILM to improve visualization and ensure complete removal in a technique called chromovitrectomy. The most commonly used dyes are indocyanine green (ICG), infracyanine green (IfCG), trypan blue, brilliant blue, and triamcinolone acetonide. Following dye injection, the ILM is grasped directly with forceps or a flap of the ILM is created and vitreoretinal forceps are used to grasp the flap (Figure 1, [5]). Pulling with the forceps in a circular motion parallel to the retinal surface, the ILM flap is extended, peeled from the retinal surface, and removed.

## 4. Common Indications

### 4.1. Macular Hole Repair

Macular holes are full thickness defects through the fovea centralis causing loss of central visual acuity, central scotoma, and metamorphopsia in affected eyes. Most of these holes are idiopathic, though trauma, inflammation, and high myopia are less common causes.

**Figure 2:**
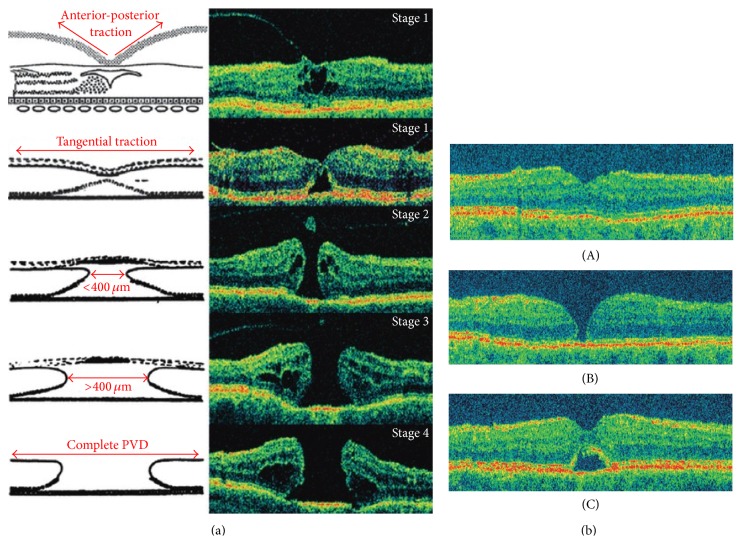
(a) Schematic and OCT representation of macular hole formation [1]. (b) Optical coherence tomography images 3 months after macular hole surgery. (A) Normal gross anatomic features with an attached fovea. (B) Flat edges with persistent neurosensory defect. (C) Contiguous foveal surface with persistent subfoveal fluid [1].

As Gass eloquently classified the progression of macular holes and, later, demonstrated with optical coherence tomography (OCT), idiopathic macular holes begin with the development of tangential traction of the prefoveal vitreous cortex [11]. This initially causes dehiscence of the outer retina in the foveal region and subsequently progresses to separation of the retinal structures and ultimately a full thickness hole at the fovea (Figure 2(a)). In a study analyzing the ultrastructure of vitreomacular interface, Schumann et al. found that fibrocellular proliferation on the vitreal side of the ILM (when present) was composed of fibrous astrocytes, myofibroblasts, fibroblasts, RPE cells, and macrophages, that collagen was invariably associated with this proliferation, and that the process was actually a secondary event in this condition [12]. Stages II–IV macular holes require closure for the best possible outcome, but it was not until 1991 that Kelly and Wendel suggested the use of surgical repair as a treatment option [9]. The literature suggests that removal of the ILM increases rates of successful hole closure by relieving prefoveal traction as well as inducing gliosis via surgical trauma [7]. Success rates in primary anatomic macular hole closure have been reported to range from 90 to 100% when vitreoretinal surgery included ILM peeling versus 60–90% when it did not include ILM peeling [1, 13–15]. Though universal better visual outcomes have been more difficult to demonstrate with adjuvant ILM peeling in macular hole surgery compared to without, a greater than 2-line improvement in vision has been reported in 60–85% of eyes [14, 15]. OCT, the gold standard diagnostic tool for retinal diseases, is tremendously useful for evaluation of macular holes preoperatively and the surgical outcomes postoperatively. Focusing on short-term follow-up, Christensen et al. analyzed data from the Copenhagen Macular Hole Study and found that 3 months after macular hole surgery OCT illustrates 3 distinct patterns in closure type, though these results were found to be the same between eyes that underwent ILM removal and those that did not (Figure 2(b), [1]). Concerning the long-term results, in a large retrospective study comparing results of surgery with and without ILM peeling after a follow-up of 18–84 months (mean 44.5 months), Brooks reported functional and visual outcomes with ILM peeling to be better than without peeling for stages II, III, and IV macular holes, acute and chronic. He reported primary hole closure without reopenings in 100% of ILM-peeled eyes and a mean postoperative vision of 20/40. The rate of reopening after primary hole closure without adjuvant ILM peeling was 25%, increasing to 100% with reoperation to include ILM peeling; mean vision remained unchanged or improved postoperatively in 78% of these reoperated eyes [13].

In a large prospective study focusing on the long-term outcomes of ILM peeling for macular holes after at least 12 months, Haritoglou et al. described promising results. The authors reported anatomic closure in 87% after 1 surgery, closure in 96% of reoperated eyes, and a median best-corrected visual acuity improvement from a median of 20/100 preoperatively to 20/40 postoperatively in 94% [16]. Following primary hole closure they did not encounter any reopenings in eyes that underwent ILM peeling as compared to variable frequencies of reopenings in eyes that did not undergo ILM peeling [17, 18]. Furthermore, the authors found that though over half of the patients developed paracentral scotomata after surgery with ILM peeling, they were subclinical in the majority of subjects and the size, shape, and density of the scotomata were unchanging in all cases [16].

As several sources have displayed favorable anatomic and functional outcomes with ILM peeling, this technique has become part of the standard of practice for vitreoretinal surgeons repairing full thickness macular holes.

### 4.2. Macular Thickness Reduction in Diabetic Macular Edema

Diabetic macular edema (DME), caused by intraretinal fluid accumulation in the macula, is the most common cause of visual impairment in diabetic patients and a major cause of legal blindness in the United States. The pathogenesis is multifactorial and includes breakdown of the blood-retinal barrier (BRB) secondary to weakened capillary intercellular tight junctions, loss of pericytes, and leukostasis in the retinal vessels and vasoactive factors such as vascular endothelial growth factor-A (VEGF-A), various growth factors, and matrix metalloproteinases. Abnormalities at the vitreoretinal interface (the posterior vitreous cortex and ILM) have also been found to promote DME. Specifically, the hyaloid becomes taut and thickened with induced cellular proliferation and production of cytokines. The fovea and the vitreous base, where the ILM is thinnest, are the points at which the posterior vitreous cortex and the ILM have the strongest attachment. Advanced Glycation End-Products (AGEs), accumulated in the posterior vitreous cortex, increase cross-linking of collagen fibrils and induce structural changes in the posterior hyaloid that strengthen vitreomacular adhesions between the posterior hyaloid and ILM. This is further aggravated by AGE receptors (RAGEs), which are attached to the footplates of the Müller cells and extend to the external limiting membrane (ELM). RAGE activation by the binding of AGEs stimulates VEGF upregulation and retinal vessel permeability, further exacerbating DME [19].

Laser photocoagulation is the standard treatment for clinically significant macular edema (CSME) as established by the Early Treatment Diabetic Retinopathy Study (ETDRS), but not uncommonly DME persists despite laser treatment [20]. Several studies have shown favorable results of pars plana vitrectomy (PPV) to address the tractional forces involved in DME [21–24], and though the role that the ILM plays in macular edema is not entirely understood, some authors have found its removal with PPV to be more beneficial than PPV alone. In an ongoing prospective study investigating the structural and visual outcomes of PPV and ILM removal in eyes with diffuse DME, Recchia et al. reported improvement in visual acuity (1 Snellen line in 100% of studied eyes) and macular thickness (20% decrease in 80% of eyes). The authors state that ILM removal ensures complete posterior hyaloid separation when PPV gives the false appearance of full separation in the event of vitreoschisis, which commonly occurs. They also suggest that as the ILM serves as a scaffold for cellular proliferation, its absence may prevent the formation of an epiretinal membrane that might otherwise occur [20].

### 4.3. Epiretinal Membrane Removal

Epiretinal membrane (ERM) is a disease of the vitreomacular interface characterized by cellular proliferation on the inner retinal surface. It is classified as either idiopathic in nature or secondary to an independent ocular pathology such as inflammation, trauma, retinal vascular disease, and surgery. Regardless of the underlying etiology, it is the contractile properties of ERM elements that have the potential to create vitreomacular traction, distort foveal morphology, and promote retinal thickening, producing symptoms of decreased visual acuity or metamorphopsia. Though ERM is relatively common among older persons, most are asymptomatic and can be managed conservatively with observation; however, development of visual disturbances or deterioration of vision warrants surgical intervention [25, 26].

The standard surgical technique for treating ERM has been established since the 1970s and entails pars plana vitrectomy with ERM removal. In general, this approach has proven to have good outcomes with the potential for few associated complications. ERM recurrence is one such complication and though uncommon, reported by Grewing and Mester to occur in approximately 12% of cases, reoperation may be indicated in cases of symptom exacerbation [27]. One reason for recurrence is thought to be secondary to incomplete removal of microscopic ERM elements, not visible with staining, that use the ILM as a scaffold for cellular proliferation. The pilot study conducted by Park et al. demonstrated that PPV for macular pucker with additional ILM peeling resulted in 0% recurrence versus 21% recurrence in those without ILM removal [28]. Shimada et al. reproduced similar results in a prospective case series aimed at determining ERM recurrence in eyes that underwent either single peeling of ERM or double peeling of ERM and ILM. The authors reported 0% recurrence rate in double-peeled eyes versus 16.3% in single-peeled eyes. Though overall there was no difference in postoperative visual acuity between the two groups, 1/3 of eyes with ERM recurrence required reoperation for impaired visual acuity, all of which confirmed via histopathologic examination the ILM to be the source of fibroblast proliferation [29].

Additional ILM peeling in surgery for ERM removal does not eliminate the potential for future ERM development, but, according to a retrospective study of 440 eyes, the recurring membrane is thin and asymptomatic. ERM does not recur often, and the need for reoperation is rare, but as of yet ILM peeling is the only measure proven to be preventative; therefore, though not a necessary component of every operation for ERM, in select cases ILM removal is invaluable in maximizing postoperative visual potential [30].

### 4.4. Myopic Macular Retinoschisis

Macular retinoschisis is a traction-induced maculopathy common among highly myopic eyes with posterior staphyloma, with manifestations including retinal thickening, formation of cystoid spaces, foveal detachment (termed myopic foveoschisis), and lamellar or full thickness macular hole. With the advent of OCT, such retinal anomalies, previously difficult to diagnose, were better characterized and discovered to be present in up to one-third of highly myopic eyes with staphylomata [31, 32].

Vitreous traction is pivotal in the pathogenesis of macular retinoschisis in highly myopic eyes, but the source of this traction is variable with etiologies including ERM, remnant cortical vitreous plaques following posterior vitreous detachment (PVD), perifoveal PVD, and a taut ILM [32–38]. Macular retinoschisis associated with vitreomacular traction increases the risk of macular hole formation and retinal detachment and necessitates surgical intervention [33, 36–38]. Several case series have reported different surgical procedures, namely, PPV, with or without gas tamponade and prone positioning, with our without ILM peeling, to be effective in promoting postoperative anatomic resolution, or retinal flattening, and improving visual acuity [32, 34, 37–39]. The specific role of the ILM in the pathogenesis continues to be investigated, but, according to VanderBeek and Johnson, there are several reports in the literature of myopic macular retinoschisis in which macular traction is not secondary to a preretinal source but rather to a taut, highly elastic ILM inducing noncompliance of the inner retina. In such cases, ILM peeling is elemental in the management of macular retinoschisis [35], but as the inciting etiology for vitreomacular traction is variable among highly myopic eyes, so too is the best surgical approach in its management.

## 5. The Complications

### 5.1. Chromophore Toxicity

Retinal toxicity can occur secondary to the specific dye used during chromovitrectomy. Indocyanine green (ICG), introduced in 2000, is a chromophore that stains the ILM secondary to its affinity for laminin and collagen type IV. Several authors have reported side effects observed with ICG use, the most common being visual field defects, reduced retinal nerve fiber layer thickness on OCT, and RPE or ganglion cell changes that manifest as abnormalities on multifocal electroretinography (mfERG), light and transmission electron microscopies, and reduced enzymatic activity [40–43]. The mechanism of injury is unclear, but the adverse effects could be related to the dose of the dye, its osmolarity, or the photooxidative qualities causing cellular damage. In an interventional consecutive case series, Tsuiki et al. postulated that postoperative visual field defects are caused by ICG toxicity via photochemical effects, that is, illumination induced chromophore excitation [44]. Based on OCT analysis, Yamashita et al. suggest ICG directly damages the retinal nerve fiber layer and is associated with postoperative visual field defects; they found a significant decrease in the measured nerve fiber layer thickness in eyes with visual field defects after ICG-assisted macular hole surgery compared to eyes without visual field defects [45]. Lai et al. assessed retinal function via mfERG performed before and after epiretinal membrane (ERM) surgery with ILM peeling using different ICG concentrations. Patients were randomized prior to surgery to receive either 0.5 mg/mL or 1.25 mg/mL of ICG staining and mfERGs done preoperatively, 3 months postoperatively, and 6 months postoperatively were compared between the 2 groups. At 3 months cone photoreceptor function, corresponding to the first negative peak (N1 or a-wave), and bipolar and Müller cell function, corresponding to the first positive peak (P1 or b-wave), were found to be significantly reduced in the group of eyes in which a higher ICG concentration was used compared to the group of eyes in which a lower concentration was used, though at 6 months no significant changes were observed in these amplitudes. The authors proposed that though there were no abnormalities of visual acuity or visual field noted, lower concentrations of ICG should be used [46].

Trypan blue (TB) is a dye that stains damaged cell membranes often used in epiretinal membrane removal in addition to ILM peeling. The formulations used in vitreoretinal surgery are low concentrations, but experimental studies have shown TB induces neurotoxic effects on retinal ganglion cells in a dose- and time-dependent manner [47, 48].

Triamcinolone acetonide (TA) is used to identify the posterior vitreous cortex, epiretinal membranes, and the ILM during vitrectomy. Conflicting evidence makes it difficult to definitively say if TA is toxic to the retina, though there are published reports of its use producing similar adverse effects to ICG. Crystal deposition secondary to TA, which aids in ILM removal, has been proposed to delay the healing process and affect macular hole closure [49]; however, in a large case series of patients who underwent idiopathic macular hole surgery with the use of adjuvant TA for ILM removal, the authors reported favorable visual outcomes and anatomical closures at a rate comparable to studies in the literature using different agents for staining [50].

Brilliant blue G (BBG) selectively binds to and stains ILM similarly to ICG and IfCG, optimizing ILM peeling. Historically found to have good clinical outcomes without evidence of toxicity on mfERG, it has widely been accepted as a good alternative dye, though its safety profile is still a matter of controversy [51]. In a case report recently published in January 2015, BBG 0.05% was used for chromovitrectomy during a PPV with ERM and ILM peeling for a patient with ERM. Following BBG injection and removal, the dye was observed to have accidentally migrated into the subretinal space in the macula, presumably through a retinal break that was not visible during the operation. Postoperative complications included cystoid macular edema observed on OCT, staining on fluorescein angiography, and amplitude reduction and implicit time increase on mfERG. It cannot definitively be concluded that these functional and anatomic changes were directly caused by macular subretinal migration of the dye, but the case report sheds light on the need for further studies to delineate the harmful effects of BBG on retinal tissue [52].

### 5.2. Damage to the Müller Cell

**Figure 3:**
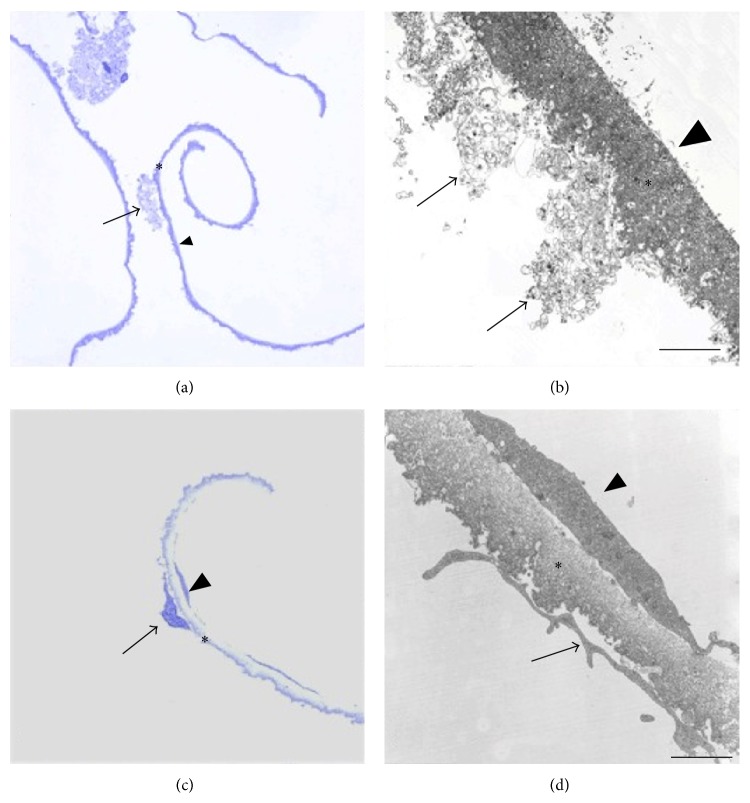
Light micrographs (LM) and transmission electron micrographs (TEM) of the ILM (asterisks) removed from eyes with diabetic macular edema ((a) and (b)) and stage IV idiopathic macular hole ((c) and (d)). The ILM is characterized by an undulated retinal side and a smooth vitreal side. ((a), (b)) Cell membrane fragments (arrow) on the retinal side of the ILM. The vitreal side of the ILM (arrowhead) is devoid of cells and collagen. ((c), (d)) LM shows a cell (arrow) with nucleus on the retinal side of the ILM. EM shows one large cell fragment (arrow) in contact with ILM and a single cell on the vitreal side of the ILM (arrowhead), which is likely a fibrous astrocyte [6].

**Figure 4:**
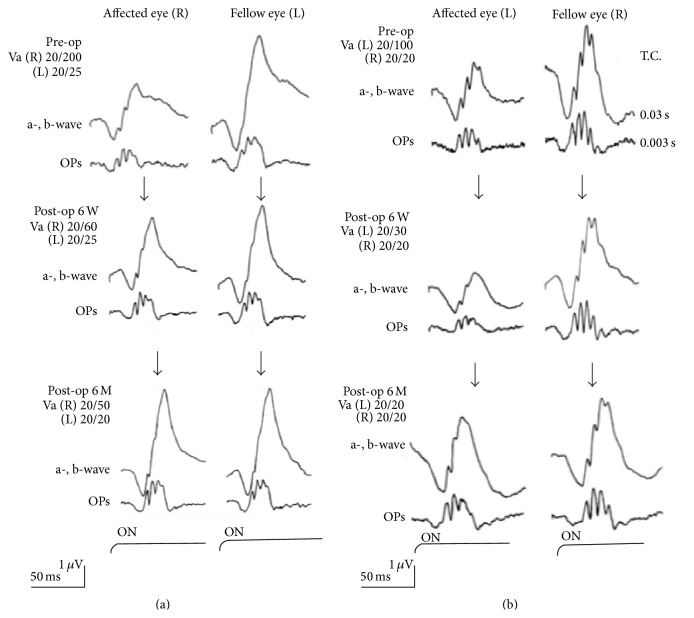
(a) Focal macular ERGs before and 6 weeks and 6 months after IMH surgery without ILM removal and the fellow eye. The b-wave amplitudes increase 6 weeks and even further 6 months after surgery. (b) Focal macular ERGs before and 6 weeks and 6 months after IMH surgery with ILM removal and the fellow eye. The b-wave amplitudes are significantly decreased 6 weeks after surgery but recover 6 months after surgery to the same level as that prior to surgery [7].

Given the close proximity of the ILM to the inner retina and its interdigitation with Müller cell footplates, it is not surprising to find retinal tissue and Müller cell debris on removed ILM specimens (Figure 3, [6] or [53]). Though there are variable amounts and sizes of such debris found on the ILM depending on the underlying disease process, one might reasonably wonder whether loss of inner retinal elements interferes with normal retinal function. Müller cells are specialized cells that contribute to retinal homeostasis [54] and they are an integral component of the ILM, contributing to formation of the b-wave on the electroretinogram (ERG). Terasaki et al. analyzed recordings of focal macular electroretinograms (FMERGs), looking at retinal physiology in the macular region of subjects undergoing ILM removal (Figure 4). The recordings demonstrated a limited and delayed recovery of the b-wave amplitude 6 months after surgery, possibly indicating dysfunction or physiologic changes of the Müller cell, though the authors did not find associated adverse postoperative visual outcomes [7]. In another study, Lim et al. also assessed whether the amount of debris on surgically removed ILM (visible on electron microscopy) affected retinal function. Implicit time (time-to-peak of the b-wave), which is a more sensitive measure of retinal damage than amplitude, was prolonged, indicating Müller cell damage and possibly subtle macular dysfunction, though final visual acuity was unaffected. Though several studies have been performed to determine the effect of Müller cell trauma on retinal function, we cannot definitively say that ILM peeling and subsequent Müller cell impairment has an overall negative outcome [53].

### 5.3. Paracentral Retinal Holes

**Figure 5:**
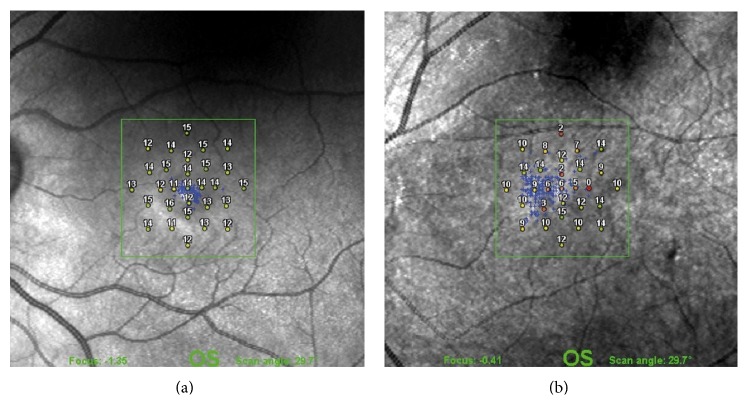
Scanning laser ophthalmoscope microperimetry after idiopathic macular hole surgery. (a) One month after surgery without ILM peeling showing normal retinal sensitivity and no deep microscotomas in the central 9 degrees of the visual field. (b) Two months after surgery with ILM peeling showing decreased mean retinal sensitivity and deep absolute and relative microscotomas in the central 9 degrees of the visual field [8].

In a case series from 2006, Steven et al. reported the formation of paracentral retinal holes following seemingly atraumatic ILM removal, observed with ICG, TB, and TA and when no adjuvant dye was used. They suggested that this postoperative finding might be a consequence of Müller cell damage causing weakening of the glial structures of the retina and ultimately hole formation. Specifically, as Müller cells remove metabolic waste products from retinal neurons, their removal in the process of ILM peeling may induce glial apoptosis and resultant retinal dysfunction. As these secondary paracentral holes always developed in the area of ILM removal, the authors discuss possibly limiting the area of retina that is peeled [55]. Since then, there have been additional reports of formation of paracentral retinal holes associated with ILM removal. A recent study was conducted investigating retinal sensitivity and frequency of paracentral microscotomas in eyes that had undergone ILM peeling compared to eyes that had not. Results of combined scanning laser ophthalmoscope (SLO) microperimetry and spectral domain OCT were used to quantify the data and demonstrated a significantly lower mean retinal sensitivity and more frequent postoperative microscotomas in eyes that underwent ILM removal (Figure 5). The exact mechanism was not elucidated, but the authors discuss that it is unlikely to be secondary to forceps-induced trauma or dye-associated toxicity. They explained that the surgeons were highly experienced and accustomed to the procedure; the diminished retinal sensitivity was diffuse and not focal (as would be expected with direct mechanical trauma); and like the previous study [55], 3 different dyes were used, none of which included ICG (the dye most strongly associated with retinal ganglion cell toxicity). Rather, the reduced retinal sensitivity development of microscotomas may be secondary to direct damage to Müller cells [8].

### 5.4. Dissociated Optic Nerve Fiber Layer

A dissociated optic nerve fiber layer (DONFL) appearance is described as arcuate retinal striae along the optic nerve fibers in the macular region, slightly darker than the surrounding retina. A retrospective case series of 91 eyes with closed idiopathic macular holes, 67 ILM-peeled and 24 non-ILM-peeled, detected a DONFL on color fundus photography in 54% (36 of 67 eyes) of ILM-peeled eyes and 0% of nonpeeled eyes. OCT was performed on 20 of the 36 eyes and all of the nonpeeled eyes and demonstrated focal dehiscence of the optic nerve fiber layer only in the 20 eyes that demonstrated DONFL. Despite these findings and previous reports of DONFL associated with ILM peeling, no functional outcomes were observed; that is, visual acuity, visual field testing, and SLO microperimetry did not show abnormalities. The authors suggest DONFL appearance may be secondary to mere shifting of optic nerve fibers, rather than deterioration, resulting from loss of Müller cell support or postoperative regenerative processes of Müller cells or astrocytes [56, 57].

### 5.5. Phototoxic Damage

Phototoxic damage to the retina can occur because of photothermal, photomechanical, or photochemical mechanisms. Photothermal damage results from prolonged exposure of the retina to a light source. Photomechanical retinal damage is a possibility if there is physical contact between the light probe and the retina. Photochemical damage results when the visible light excites endogenous or exogenous chromophores. The endogenous chromophores excitable by visible light wavelengths are the photoreceptor pigments, as well as the melanin and lipofuscin of the RPE. ICG is an example of an exogenous chromophore excitable by visible light. Chromophore excitation produces reactive oxygen species, which cause lipid peroxidation and ultimately destroy cell membranes [1].

## 6. Conclusion

Though the ILM is integral to the histogenesis, structure, metabolism, and function of the retina, its detrimental role in inducing or exacerbating traction in various vitreoretinal diseases has made its removal in the treatment of traction-induced maculopathies logical and absolutely necessary, so much so that its indications have extended from the idiopathic full thickness macular hole from which it was born to include several other conditions that have an element of prefoveal traction. ILM peeling has revolutionized and become a vital component in vitreoretinal surgery as it has repeatedly been shown to be safe and effective in improving anatomic and functional outcomes across a range of retinal diseases, but the technique is not resistant to causing perioperative retinal damage and several authors in the literature have reported objective abnormal findings postoperatively. Despite its widespread acceptance and high safety profile, it is of paramount importance to always be aware of the possible deleterious consequences ILM peeling can impose, because as routine as the technique has become for the field and for the surgeon, it indeed is not routine for the patient.
